# Towards a strategic alignment of public health and primary care practices at local levels – the case of severe and enduring mental illness

**DOI:** 10.1080/17571472.2018.1437070

**Published:** 2018-03-06

**Authors:** Ricky Banarsee, Cornelius Kelly, Austen El-Osta, Paul Thomas, Chris Brophy

**Affiliations:** a Public Health England, London, UK; b Central and North West London Foundation NHS Trust, London, UK; c Self Care Academic Research Unit (SCARU), Imperial College, London, UK; d London Journal of Primary Care, London, UK; e T7 Associates, London, UK

**Keywords:** Community-oriented integrated care, new care models, public health, partnership, community hubs, mental health

## Abstract

The rapidly increasing number of people who have long-term conditions requires a system of coordinated support for self-care throughout the NHS. A system to support self-care needs to be aligned to systems that support shared-care and community development, making it easier for the multidisciplinary teams who provide care to also help patients and populations to help themselves. Public health practitioners need to work closely with clinicians to achieve this. The best place to coordinate this partnership is a community-based coordinating hub, or local health community – a geographic area of about 50,000 population where different contributions to self-care can be aligned. A shared vision for both health and disease management is needed to ensure consistent messaging by all. A three tier system of shared care can help to combine vertical and horizontal integration. This paper uses severe and enduring mental illness as an exemplar to anticipate the design of such a system.

## Why this matters to me

London Journal of Primary Care (LJPC) publishes articles on the multi-dimensional aspects of primary care that make it so human and vibrant. Journal members share ideas and experience to co-produce papers that point to good policy for community-oriented integrated care and health promotion [1,2,3]. We hope that this paper will stimulate general practice and more broadly primary care, to pilot collaborations with public health, local authorities and other organisations.

## Key message

What the reader might learn from the paper.

In order to improve the health of patients with long-term conditions such as severe and enduring mental illness, public health and local authorities should work in partnership with clusters of general practices to develop communities for health, within which self-care, shared-care and participation in local projects is a cultural norm.

## Partnership between public health and primary care is essential to support self-care

Muir Gray has argued that:

self-care is the most important type of care…even when people are receiving excellent technical care from a generalist, specialist or super-specialist, much depends on what they will do for themselves. If people in their 70s, 80s and 90s gave up caring, then the National Health Service (NHS) would collapse tomorrow. [5]

Self-care is especially needed to contain the cost of long-term conditions [6]:

• Nearly 70% of NHS spending on primary care and hospital care supports people with long-term conditions.
• People with long-term conditions use 50% of all GP appointments, 64% of all outpatient appointments and 70% of all inpatient bed days.
• Good self-care could reduce A&E attendances by 15%.

More than 30% of people in the UK have a long-term condition, rising to over 60% in those aged over 65. These people DO want to self-care. 82% of people with a long-term condition said they already play an active role in their care; over 90% wanted to be more active self-carers; over 75% said that if they had guidance and support from a professional or peer they would feel more confident about self-care [7].

People self-care by doing things that prevent problems or address them at an early stage. They: (a) Monitor their conditions and update their care plans, (b) Access information, courses and advice, (c) Seek help in a crisis, (d) Accept opportunities for social support, self-development and healthy lifestyles.

To make self-care happen at scale, public health practitioners, who focus on prevention, need to work closely with clinicians, who focus on treatment. This is the conclusion of the UK Academy of Medical Sciences that suggested a radical rethink of the public health role to address the ‘*complex array of interlinking factors that influence the health of the public’.* The report recommends:

Better alignment between public health and clinical practice…. if we are to achieve the necessary shift to prevention (p5). [8]

*Provider Voices* (the membership organisation and trade association for the NHS acute, ambulance, community and mental health services) explains that many people in society, not merely clinicians and public health practitioners, need to take their part in public health. They state: ‘*Public health is everybody’s business, it operates at every level and matters to national government as much as individuals’*. In order to explore how to achieve this in the NHS, Provider Voices interviewed twelve experts who reflected the views of commissioners, providers, operating nationally, regionally and locally [9].

They concluded:

• Structurally – maintain and enhance the focus on the public health role of Sustainability & Transformational Partnerships and Accountable Care Organisations.
• Financially – reverse the cuts to local government public health budgets and shift payment systems so that the incentive is to prevent rather than cure.
• Clinically – reinstate a strong and strategic role for public and population health clinicians in provider organisations which will benefit all other parts of the public health system.
• Culturally – bring about a change in mind-set across the NHS that is focused on public health and its role in empowering individuals to look after their health.
• As the public sector – continue on the journey of influencing fundamental determinants of health and health inequalities.

They suggest that Accountable Care Organisations should be the ones to lead the alignment of public health and clinical practice in the NHS. Having said that, it will need an alliance of forces, so anyone who can should lead it, perhaps as an exercise in shared leadership. An alliance of forces is most likely to gain the advantages of efficiency and quality from such alignment and keep in mind the array of things that affect health that can be invisible from single perspectives. It could also use existing skills and models in new ways. For example, local authorities have considerable experience of building communities and this could add to the skills brought by public health and primary care to provide efficient, locally-relevant models.

In this paper we argue that policy should systematically develop geographic localities of about 50,000 population, with the intention of developing, within them, systems for self-care, shared-care and community development. These should be facilitated by public health, local authorities and primary care practitioners working in partnership. In the long-term this could lead to a grand alliance for health and care throughout society. It could build from the present policy to make care plans for people with long-term conditions.

## Geographic localities as community-based hubs of coordination

Intended to reduce costly hospital admissions of patients with long-term conditions, in 2015 general practitioners became funded to make *Care Plans* for 2% of their patients most at risk of hospital admission. At about the same time, general practices started to cluster into geographic localities of about 50,000 population to coordinate care and lead collaborative improvements. These areas were termed *Health Networks* when considering medical care (e.g. Care Plans) and *Local Health Communities* when considering broader aspects of health (e.g. Healthy Environments).

Localities are good places to think about *environments for health* [10]. In specialist places, like hospitals, the focus is on the reason for being there – usually treating a specific disease. In localities, where people live out their lives, it is as least theoretically possible to facilitate a grand alliance for self-help, horizontally building teams and communities for care and health promotion, and vertically connecting with specialists through care pathways. The World Health Organisation (WHO) calls these ‘community-based hubs’. A 2008 WHO report advocated ‘[community-based hubs that enable…] reorganisation of health services as primary care….coordinator of a comprehensive response at all levels… [and] secures healthier communities, by integrating public health actions with primary care and by pursuing healthy public policies across sectors’ [11].

This introduces a new role into the NHS – facilitating the development of *local health communities.* Community development is not on the curriculum of doctors or healthcare managers, so there is much to be learned. There is also little infrastructure to support it, and no obvious funding. So we should go at this thoughtfully, step by step, teasing out the practical and financial implications with a small number of conditions before going to scale.

In the rest of this paper we consider the case of severe and enduring mental illness helps us to imagine how to strategically align the roles of public health and primary care to create a healthy society.

## The case of severe and enduring mental illness

*Severe and enduring mental illness* (SEMI) affects about 1% of the population. It includes illnesses like psychosis and major depression that require life-long care. Here we use SEMI to consider the design of a system that supports self-care within local health communities. SEMI is a useful exemplar of a long-term condition because a care planning approach has been used for a long time and many patients have already been discharged from Mental Health Trusts - so strategy for shared-care and self-care already exists. Also SEMI makes obvious something that is less obvious in other conditions - when patients are ill they are less able to care for themselves. Indeed they may be unable to see that they are unwell and have to trust judgements of others to accept that they are in trouble. Further, their behaviour can be aggressive and defensive, making others reluctant to help. A system of support for self-help must therefore work hand in hand with a system for shared-care - teams of trusted carers who patients give permission to act when they are unwell.

In 2015 a LJPC Think Tank highlighted the scale of mental health problems [12]:

• 10% of children and 17.6% of adults have a mental disorder at any one time
• Half of common mental disorders, mostly depression and anxiety, remain undiagnosed
• Mental disorders are the leading cause of sickness absence in UK

This means that for every person with SEMI there are ten others who have a different mental health illness, and 30 who have a long-term condition of some kind. They all need self-help. They all need shared-care. The NHS needs to support self-help and shared-care for about one third of the population. If we can agree the principles in one condition, we may be able to apply them to others, and develop, at scale, combined self-care, shared-care and ongoing nurture of local health communities.

## Obstacles to partnership between public health and primary care

On the face of it, it is obvious that public health, local authorities and primary care should work together to design and maintain a system to facilitate self-help, since their skills and knowledge are mutually enhancing. They will need to work with specialists of various kinds to treat diseases (e.g. diabetes, cancer, respiratory and heart conditions…). Life-style and community development of course. Then there is housing, jobs, social services, occupational health, support for relationship breakdown, community policing (the police often take a “pastoral” role when people with a mental health crisis end up in the criminal justice system), and multiple voluntary sector organisations.

However, we see three natural obstacles to primary care and public health collaboration that might obstruct success:

(1) Different constituencies

*Obstacle*: Public health serves whole populations. Primary care clinicians serve individual patients on a practice ‘list’. They need a shared set of constituents to synchronise their work.

*Possible solution*: Geographic localities can provide a shared set of constituents that both public health and general practices can relate to. Each local health community can be seen as a ‘cell’ in the ‘body’ that is healthcare, and also in the ‘body’ that is society.

(2) Different aims

*Obstacle*: Public health emphasises pro-active pursuit of health, whereas clinicians emphasise reactive treatment of diseases. They need a shared vision for both personal care AND whole society health to feel that they are working to the same end.

*Possible solution*: People are likely to agree that health combines feeling good and functioning well. In the language of MacIntyre it means developing a *positive narrative unity* - continually moving forward one’s life story in positive, coherent ways [13]. In the language of Antonovski it means being ready, willing and able to rise above adversity [14]. Good health, and good mental health, are active things; they allow people to engage positively in the world. Illness can challenge health, but not define it.

(3) System fragmentation

*Obstacle*: In the NHS, public health and clinicians have always worked in silos, inhibiting joint working. Transactional interactions between them are not enough since high quality team-working requires co-creative inter-activity.

*Possible Solution*: Learning organisation principles can build high-performing teams through collective reflection and action. These can be used in local health communities through cycles of learning and change that at the same time develop the broader community. Learning organisation principles have been used in organisational development in the NHS [15], but momentum slowed when the ‘New Public Management’ emerged [16], with its focus on individualism and mechanical efficiency. A learning organisation/learning communities approach has been moved forwards in other countries using the language of *Community Development Agencies*, as an antidote to the mechanistic (and expensive) model of *Health Maintenance Organisation* [17].

A model of shared-care that fits with the vision expressed here for locality-based support for self-care is the Ealing ‘Three Tier’ model of shared-care for diabetes [4]. This could be easily adapted to develop shared care for SEMI. This model includes a specialist-led ‘Diabetes Intermediate Care Clinic’ that links to geographic localities (termed Health Networks) of about 50,000 population. As well as seeing patients, clinic staff provide decision-support and educational updating to the (5–12) general practices in the Health Network, each of which runs its own diabetes clinic. A Health Network multidisciplinary leadership team holds meetings; at these meetings different disciplines work together to develop Care Plans (for diabetes and many other conditions), evaluate the system and pilot improvement projects. Throughout Ealing as a whole the work of seven Health Networks, each of which is undertaking this kind of activity, is integrated through a supportive Facilitation Team, a strategic Oversight Team and a Leadership Course where various leadership teams learn from and with each other about how to sustain progress.

## Elements of a system to support self-care for severe enduring mental illness

These three components – shared vision for health, a three tier system for shared-care and systematic development of local health communities – could provide the environment that enables public health, local authorities, primary care and mental health practitioners to collaborate to support self-care and shared-care for SEMI (and all other long-term conditions). Each Health Network would need to maintain:

• *Care plans*: Patients, mental health and primary care practitioners would agree these, listing the care team, annual goals and self-care. The plan would be accessible by relevant agencies including Out of Hours and ambulance services
• *Crisis intervention*: To be triggered by the patient, care team, or others as needed
• *Education*: Information, courses and telephone advice for patients and carers
• *Drop-in centres*: Includes opportunities for self-development, relationship-development and healthy life-styles
• *Local health community workshops*: Regular meetings for clinicians, patients and policy-makers to evaluate the system and pilot improvements.

Various models of community building can be piloted to enhance the basic model. For example, the work of Hans Kai in Canada. The Hans Kai model supports a cooperative of patients who are hosted by a community health organisation; they support each other about a range of things, including healthy eating, exercise and social support [18].

Other mechanisms to support such complex, multi-agency collaboration have been piloted [19] including:

• *Seasons of Care, Health Promotion and Participatory Action Research* to engage large numbers of organisations in annual cycles of inter-organisational learning and change, coordinating media campaigns, Care Plan updates and services like ‘flu vaccinations and self-development
• *Live Manuals* where leadership teams put information, including educational updates, improvement projects and descriptions of what different people do
• *Large Group Events* that help people to creatively interact and build communities for health

## Discussion

There is growing recognition that everyone needs to contribute to self-care and shared-care. Geographic ‘community hubs’ provide an opportunity for joint working between public health, local authorities, primary care clinicians and mental health specialists to practically achieve this. Long-term conditions such as SEMI provide specific conditions to work with. Other initiatives, including Community Education Providers Networks, could work to the same geographic boundaries to enhance overall effect. Clusters of such localities could make up larger entities that, for example for commissioning. Ensuring that these hubs are effective will require a shared vision for health, a three tier system for shared-care and systematic development of local health communities. Each local health community needs a set of services for patients to access, and annual cycles of collaborative learning and coordinated change to facilitate ongoing improvement of the whole system.

There are signs in the UK that policy is moving in a way to support this kind of complex, collaborative activity:

• NHS policy described in the *Five Year Forward View* recognises a move towards shared care and self-care stating:
  many people wish to be more informed and involved with their own care, challenging the traditional divide between patients and professionals, and offering opportunities for better health through increased prevention and supported self-care [20].
• NHS policy for mental health research describes the need for practitioner-patient/user collaboration relevant to the context, advocating ‘*life course approach’, ‘patient & public involvement’* and *‘user*-*led’ translational research* [21].

This move towards collaboration for long-term success chimes with a growing appreciation of the value of ‘Third Way’ politics [22]. Instead of lurching from one side of the boat to another at every policy revision, we need to keep everyone on board by enabling individual creativity within a broad vision for health. 50 ‘New Care Models’ in the UK, termed ‘Vanguard Sites’, are presently exploring ways to do this [23]. They may wish to consider the ideas in this paper.

## Governance

WeLReN CiC and LJPC Board provided overview and critique of this paper.

## Evidence of permissions to publish other people’s work

Not applicable

## Funding

NHS Public Health funded the lead author.

## Disclosure statement

No potential conflict of interest was reported by the authors.
